# Therapeutic potential of mesenchymal stromal cells for hypoxic ischemic encephalopathy: A systematic review and meta-analysis of preclinical studies

**DOI:** 10.1371/journal.pone.0189895

**Published:** 2017-12-19

**Authors:** Jamie Archambault, Alvaro Moreira, Dawn McDaniel, Lauryn Winter, LuZhe Sun, Peter Hornsby

**Affiliations:** 1 Department of Pediatrics, Division of Neonatology, University of Texas Health-San Antonio, San Antonio, Texas, United States of America; 2 Department of Cell Systems and Anatomy, University of Texas Health-San Antonio, San Antonio, Texas, United States of America; 3 Department of Cellular and Integrative Physiology, Barshop Institute for Longevity and Aging Studies, University of Texas Health-San Antonio, San Antonio, Texas, United States of America; Fraunhofer Research Institution of Marine Biotechnology, GERMANY

## Abstract

**Introduction:**

Neonatal hypoxic ischemic encephalopathy (HIE) is a devastating neurologic condition with high mortality rates and long-term complications for surviving infants. Mesenchymal stem/stromal cells (MSCs) have emerged as novel therapeutic agents with promising results in experimental studies of HIE. The purpose of this study is to (a) methodically review the current preclinical literature describing MSC therapy in animal models of HIE, (b) quantify the effect size in regards to functional neurologic outcome, and (c) identify research gaps/limitations that should be addressed prior to future preclinical and clinical studies.

**Methods:**

Adhering to the Systematic Review Protocol for Animal Intervention Studies, a systematic search of English articles was performed. Eligible studies were identified and data regarding study characteristics and outcome measures was extracted. After quality assessment, meta-analysis and meta-regression were performed to generate random effect size using standardized mean difference (SMD). Funnel plots and Egger’s tests were utilized to evaluate for the presence of publication bias.

**Results:**

A total of 19 studies met inclusion in the current systematic review. Meta-analysis revealed that MSCs have a significant positive effect on neurobehavioral outcome following HIE injury. Sensorimotor function was improved by 2.25 SMD (95% CI; 2.04–2.46) in cylinder rearing and 2.97 SMD (95% CI; 2.56–3.38) in rotarod. Likewise, cognitive function was improved by 2.76 SMD (95% CI; 2.53–2.98) on the water maze and 2.97 SMD (95% CI; 2.58–3.35) in object recognition. Stratification demonstrated an increased effect size depending on various study characteristics.

**Conclusions:**

Overall, these results suggest a promising role for MSCs in preclinical studies of HIE. MSC treatment demonstrates improved functional outcomes that are encouraging for future translational studies. While risk of bias and heterogeneity limited the strength of our meta-analysis, our results are consistent with those seen in this field of research.

## Introduction

Neonatal encephalopathy is a devastating constellation of symptoms resulting from widespread central nervous system dysfunction. Affected infants are born with low Apgar scores, severe acidemia, evidence of acute brain injury on neuroimaging, and multi-system organ failure [1]. Hypoxic ischemic encephalopathy (HIE) is a specific subset of neonatal encephalopathy caused by ischemic/anoxic brain injury in the perinatal period. The incidence of HIE ranges from 1.0 to 8.0 per 1,000 live births [2]. If not fatal, the vast majority of affected neonates will demonstrate long-term neurologic deficits such as hearing and visual impairment, developmental delay, cerebral palsy, and/or seizures [3–5].

The neurologic insult is caused by maternal, placental, or fetal conditions that result in impaired tissue perfusion, leading to a reduction in oxygen and nutrient transport to tissues [6]. On a cellular level, decreased oxygen delivery results in a primary energy failure that necessitates anaerobic metabolism. Decreased activity of the ATP-dependent Na^+^/K^+^ pump leads to intracellular Na^+^ accumulation, cytotoxic edema, and membrane depolarization that triggers release of the excitatory neurotransmitter glutamate [7]. Glutamate binds at postsynaptic N-methyl-D-aspartate (NMDA) and α-amino-3-hydroxy-5-methyl-4-isoxazolepropionic acid (AMPA) receptors and increases cytosolic Ca^2+^ [7]. Through a cascade of events, mitochondrial dysfunction and accumulation of reactive oxygen species ultimately cause necrotic and apoptotic cell death in the affected region [8,9].

The mainstay of treatment for HIE is therapeutic hypothermia, which is neuroprotective rather than neurorestorative. Literature suggest that cooling the deep brain structures to 32°C to 34°C within the first six hours of primary injury can prevent the sequlae leading to oligodendrocyte loss [10,11]. Although this therapy has been shown to improve survival and neurodevelopmental outcome, the neuroprotective response is limited by timing of initiation and severity of encephalopathy [12–14]. Duration of therapy has also been found to play a significant role in treatment outcome. Current standard of care in neonatology recommends a cooling period of 72 hours for maximum efficacy. In aged brains, hypothermic treatment for less than 48 hours actually accelerates inflammation, resulting in a larger overall area of infarct and edema [15]. Furthermore, therapeutic hypothermia of any duration has been associated with pancytopenia, coagulopathy, worsening acidosis, electrolyte imbalance, pulmonary hypertension, and arrhythmias [16]. Despite advancements in care, the overall mortality and burden of disability remains high. Thus, there is significant need for novel therapeutic measures in this area of neonatology.

In the past decade, mesenchymal stem/stromal cells (MSCs) have emerged as a promising new therapy in the management of HIE. MSCs are of particular clinical interest because they pose no ethical issues and can be harvested from many sources [17]. MSCs are non-immunogenic, easy to proliferate, and have the unique ability to differentiate into multiple cell types, including neurons [17,18]. They migrate to sites of inflammation where they exert anti-inflammatory effects and reduce reactive oxygen species [19]. Adult clinical trials have already demonstrated the regenerative potential of MSCs in orthopedic injury, cardiovascular disease, liver disease, autoimmune conditions, and graft-versus-host disease [20–24].

Preclinical studies using animal models of HIE have demonstrated that MSCs improve functional outcome in treatment groups. The therapeutic potential of MSCs for neurologic disease lies in their capacity to restore cellular energy, blunt the inflammatory response, promote neurogenesis, and enhance angiogenesis in the hypoxic region [19]. Investigators have recently demonstrated feasibility and safety in the use of autologous cord blood for treatment of human infants with HIE, paving the way for future randomized controlled trials [25]. Despite this progress, there has been no effort to synthesize the current literature exploring stem cell therapy for the treatment of HIE. The focus of our paper will be to systematically examine the efficacy of MSCs as a therapeutic agent in preclinical models of HIE. The results of this study are intended to help guide methodology of future preclinical studies and clinical trials in this area of research.

## Methods

Our methods adhere to the guidelines established by the Systematic Review Centre for Laboratory Animal Experimentation (SYRCLE) and are described in S1 Table [26]. The SYRCLE protocol was recently published as a high-quality and standardized method of analyzing preclinical animal intervention studies. Our protocol was registered through the Collaborative Approach to Meta-Analysis and Review of Data from Experimental Studies (CAMARADES) on January 23^rd^, 2017.

### Literature search

To summarize, we performed a literature search using MEDLINE’s database PubMed, Web of Science, Google Scholar, and Cumulative Index to Nursing and Allied Health Literature (CINAHL) through June 9^th^, 2017. Search terms included “mesenchymal stem cells,” “hypoxic ischemic encephalopathy,” “neonatology,” “preclinical,” and any of their synonyms (refer to S2 Table). Duplicate studies were manually removed from the search results prior to the screening process. Screening by title/abstract and subsequent full-text review were conducted independently by two investigators (JA and AM). A third investigator (DM) was consulted to resolve differences of opinion in either phase. Reference lists of included studies and relevant reviews were hand-searched in an effort to obtain additional studies for inclusion.

### Inclusion and exclusion criteria

In the current study, HIE is defined as an acute interruption of blood flow, oxygen, and nutrients to the brain. In preclinical models, HIE is typically induced via ligation or occlusion of the carotid artery, middle cerebral artery, maternal uterine artery, or umbilical cord. Studies were included if they reported the effect of MSC intervention on functional neurologic outcome in validated preclinical *in vivo* models of neonatal HIE. For rodents specifically, a neonatal model refers to less than ten days of age [27]. In our review, a MSC will be defined per the International Society for Cellular Therapy (ISCT) [28]. Papers with MSC intervention were included regardless of dosage, timing, frequency, and source, but were excluded if the investigators used modified MSCs or those combined with other therapies. However, labeled MSCs (green fluorescent protein, iron oxide particles, etc.) for tracing and locating distribution of cells were allowed. The treatment group received MSCs following HIE induction, while the control group underwent the HIE injury but was treated with vehicle/placebo (e.g. normal saline, phosphate buffered saline).

### Primary and secondary endpoints

We defined our primary endpoint as functional neurologic outcome, which is reported through cognitive or sensorimotor testing following induction of HIE. We included cognitive (e.g. water maze, object recognition, open field) and sensorimotor (e.g. cylinder rearing, rotarod, adhesive removal) tests that are routinely used in preclinical studies of neurologic insult. Studies were excluded from the selection process if data pertaining to our primary outcome could not be obtained during the data extraction phase of our study.

Our secondary outcome of lesion size was not required for inclusion, but is reported in many of the selected studies. Lesion size is described as evidence of structural improvement in either neuroimaging or histologic studies, particularly those utilizing microtubule-associated protein 2 (MAP2) and myelin basic protein (MBP), which are markers for gray and white matter, respectively.

This review presents the results of the primary endpoint analysis, while results from the secondary endpoint analysis will be reported in a future paper.

### Data extraction

Data was collected independently by two investigators (JA and AM) and compared for accuracy. A third investigator (DM) was consulted to resolve differences of opinion. Extracted data included general study design (objective, sample size, HIE model, anesthesia), animal characteristics (animal model, gender, age, immune status), intervention characteristics (source, dose, delivery, timing, frequency), and outcome measures relevant to our primary endpoint. Original data, including mean with standard error of the mean, was gathered from graphs and plots using GetData graph digitizer version 2.26 when exact values were not available from the article. In studies that reported multiple variations (dose, day, frequency) of the intervention, we regarded these results separately. We also collected the MSC characterization criteria (plastic adherence, differentiation, and positive/negative surface markers) that were reported in each study.

### Risk of bias

Risk of bias for each experiment was assessed independently by two investigators (JA and AM) based on SYRCLE’s Risk of Bias tool [29]. A third investigator (DM) was consulted to resolve differences of opinion. The SYRCLE tool contains ten assessment domains related to selection, performance, detection, attrition, and reporting biases. Each domain was scored as low, high, or unclear risk of bias based on signaling questions provided by the tool. A response of “yes” indicates low risk of bias and a response of “no” indicates high risk of bias. Studies that did not explicitly state their methods were scored as “unclear.”

### Data analysis

Meta-analysis was conducted using a random effects model to generate forest plots. The estimated effect size of MSCs on functional neurologic outcome after HIE was determined using standardized mean difference (SMD) and a 95% confidence interval (CI). SMD, an ideal measure for continuous data, is calculated by dividing the mean difference in each study by that study’s standard deviation. Stratified effect size was measured individually for sensorimotor function and cognitive function. Pooled data from all neurobehavioral studies was also examined to determine overall effect size.

Statistical heterogeneity between studies was calculated using the I^2^ metric, with I^2^ >50% suggesting obvious heterogeneity. Potential sources of heterogeneity, if significant, were further investigated by meta-regression and subgroup analysis. The presence of publication bias was evaluated using funnel plots and Egger’s tests. Funnel plots were visually assessed for asymmetry. For Egger’s tests, *p* < 0.05 was considered significant to confirm the presence of small study size.

All statistical analyses were performed using the program STATA version 13 (College Station, TX, USA). All statistical tests were two-sided and difference was considered significant when *p* < 0.05.

For certain measures of functional outcome (staircase test, electrical measures), either incomparable data points or a low number of comparisons prevented quantitative analysis. Rather than incorporating this data into the meta-analysis, we have provided a narrative summary of significant results.

## Results

### Study selection

**Fig 1:**
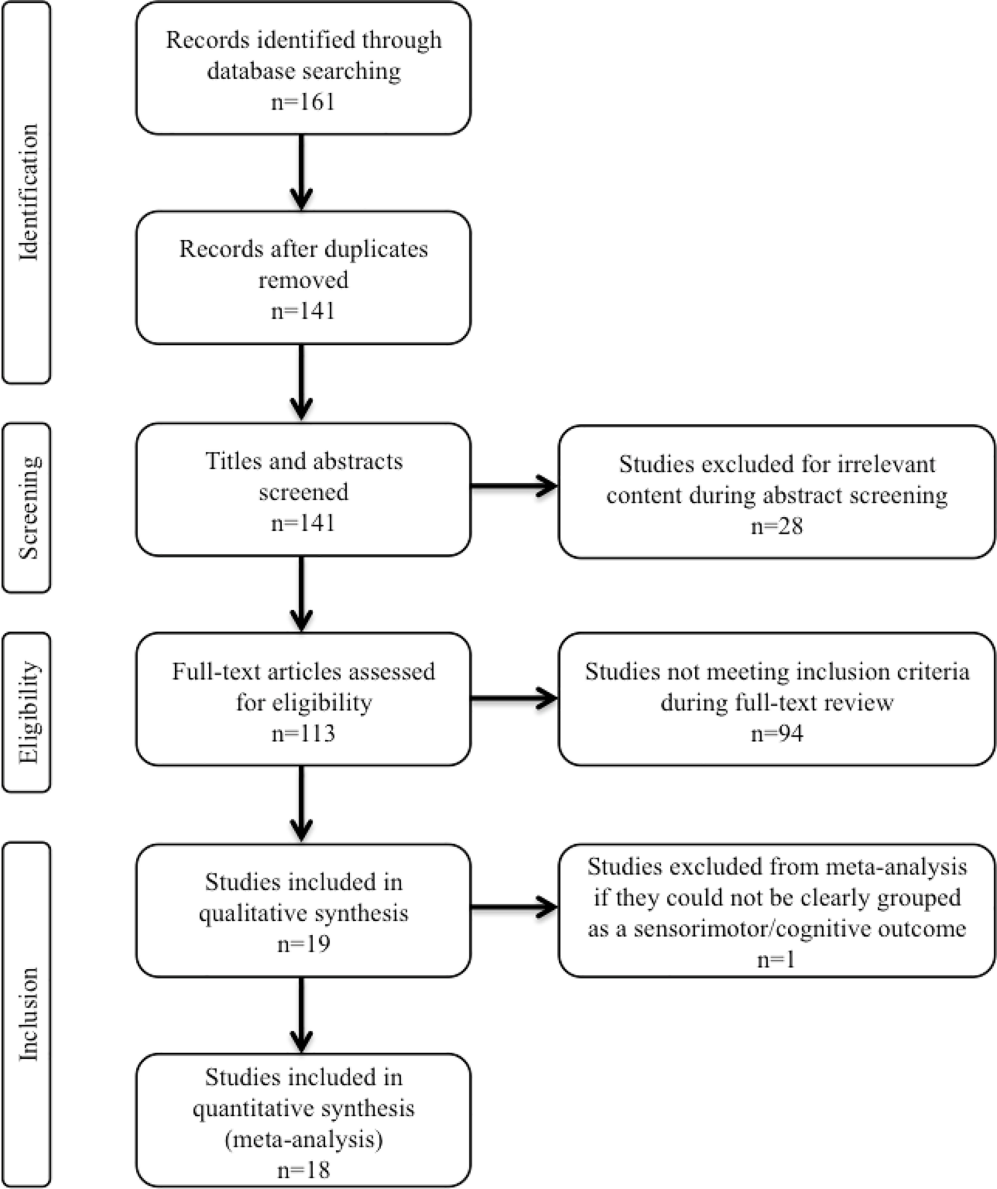
Flow diagram demonstrating study selection process.

Our literature search generated 161 results based on the utilized search terms. A total of 141 studies remained after duplicates were manually removed. After preliminary screening by title and abstract, 113 studies investigating the therapeutic potential of stem cells in HIE were isolated for full-text review. From this, 19 publications met the pre-defined eligibility criteria and reported our primary endpoint of functional neurologic outcome (Fig 1). All of these studies were reported in the review, however only 18 were included in the meta-analysis.

### Study characteristics

All studies included in this review were published between the years 2010 and 2016. Among the included articles, nine are from Netherlands, seven are from China, two are from South Korea, and one is from New Zealand. Relevant characteristics are described briefly in Table 1 and more thoroughly in S3 Table. Rodents were the most commonly studied animal model, with 58% (n = 11) of studies using either Sprague-Dawley or Wistar rats and 37% (n = 7) of studies using C57Bl/6 mice. One study investigated the effects of seizure burden on ovine fetuses, but was not included in the final meta-analysis. The gender was not reported in 68% (n = 13) of studies. All studies induced HIE by postnatal day ten, the rodent age consistent with a human neonate. Carotid artery occlusion followed by hypoxia was the HIE model employed in 79% (n = 15) of studies. The remaining studies utilized either middle cerebral artery occlusion or umbilical cord occlusion. Intracerebral injection was the most common route of delivery with doses ranging from 100,000 cells to 3,000,000 cells. Regarding intervention timing, 43% (n = 9) of studies administered MSCs within 72 hours of HIE. MSCs were administered after day three but before day ten in 33% (n = 7) of studies. Multiple doses were administered in 24% (n = 5) of studies, with most studies giving only two doses.

**Table 1:** Summary of study characteristics.

Animal characteristics	n (%)
*Animal type*	
Rat	11 (58)
Mouse	7 (37)
Sheep	1 (5)
*Rat (n = 11)*	
Sprague-Dawley	9 (82)
Wistar	2 (18)
*Mouse (n = 7)*	
C57Bl/6	7 (100)
*Gender*	
Male	3 (16)
Mixed	3 (16)
Not reported	13 (68)
*Age*	
Fetal	1 (5)
3 days	1 (5)
7 days	7 (37)
9 days	7 (37)
10 days	2 (11)
Not reported	1 (5)
**Mesenchymal stromal cell characteristics**	
*Source*	
Bone marrow	10 (53)
Umbilical cord	5 (26)
Placenta	1 (5)
Not reported	3 (16)
*Origin*	
Allogeneic	11 (58)
Xenogeneic	8 (42)
*Dose (n = 24;>1 dose per study included)*	
≤250,000 cells	9 (37)
>250,000 cells—≤500,000 cells	5 (21)
>500,000 cells—≤1,000,000 cells	5 (21)
>1,000,000 cells	5 (21)
**Experimental characteristics**	
*HIE model*	
Carotid artery occlusion + hypoxia	15 (79)
Middle cerebral artery occlusion	2 (11)
Umbilical cord occlusion	1 (5)
Not reported	1 (5)
*Delivery*	
Intracerebral	9 (47)
Intranasal	5 (26)
Intravenous	2 (11)
Other	3 (16)
*Timing after HIE injury (n = 21;>1 administration per study included)*	
≤72 hours	9 (43)
>72 hours	7 (33)
Multiple doses	5 (24)
**Survival periods**	
*Duration of study*	
≤30 days	11 (58)
>30 days—≤ 3 months	7 (37)
> 1 year	1 (5)
*Mortality rate after HIE induction*	
<10%	3 (16)
10%	6 (31)
>10%	3 (16)
Not reported	7 (37)

Functional neurologic outcome was assessed in all studies per our inclusion criteria (S4 Table). The cylinder rearing and rotarod tests were the most frequently used methods of assessing sensorimotor function, while the water maze and novel object recognition tests were the most common for cognitive function. Composite scores such as the modified neurological severity score (mNSS) and Longa score were only mentioned in two studies. Seizure burden and field excitatory postsynaptic potentials (fEPSPs) were measured in one and two studies, respectively. However, these measures were not included in the meta-analysis.

### MSC characteristics

S5 Table summarizes the characterization of the cells used in the HIE experiments. Using the ISCT criteria, 74% (n = 14) of the publications specified that the utilized cells were indeed MSCs [28]. Plastic adherence was reported in 68% (n = 13) of these studies. The ability of MSCs to differentiate into various cell lineages (e.g. adipocytes, chondrocytes, osteocytes, fibroblasts) was reported in 37% (n = 7) of studies. Positive and negative markers specific to MSCs were confirmed in 79% (n = 15) of studies. However, five of the studies that reported negative markers identified them simply as myeloid and hematopoietic cell lineage specific antigens, rather than naming specific markers.

Bone marrow was the most common source of MSCs, followed by umbilical cord and placenta. Fifty-eight percent (n = 11) of studies performed allogeneic transplant, while 42% (n = 8) of studies performed xenogeneic transplant. Dulbecco’s Modified Eagle Medium +/- Fetal Bovine Serum were used to expand cells in the majority (74%) (n = 14) of studies. Six of these studies also included an antibiotic solution in their culture media. A passage number less than five was reported in 37% (n = 7) of studies, with an additional two studies reporting passage number less than ten. Approximately half (n = 10) of the publications utilized MSCs purchased or supplied by another commercial manufacturer.

### Risk of bias

Risk of bias was assessed using the SYRCLE Risk of Bias Tool for all 19 studies that met inclusion criteria for our review (S6 Table) [29]. None of the experiments were judged as low risk of bias across all domains. All studies reported similar experimental and control groups at baseline, which reduces the risk of selection bias based on animal characteristics. Despite stating that allocation of subjects to experimental and control groups was random, none of the studies explicitly described a method of random sequence generation. For this reason, risk of bias in the sequence generation domain was judged as “unclear” in all studies. Similarly, only 5% (n = 1) of studies adequately described the method used to conceal allocation. None of the studies stated that animals were randomly housed, but 11% (n = 2) of studies endorsed blinding of caregivers and investigators from knowing which intervention each animal received. Only 5% (n = 1) of studies reported random outcome assessment, but 26% (n = 5) of studies documented blinding of the outcome assessor. Using the signaling questions provided, all studies were scored as low risk of attrition and reporting bias. Furthermore, we did not identify any additional sources of bias not already covered by the SYRCLE Risk of Bias Tool, such as industry funding, conflict of interest, or failure to publish in a peer-reviewed journal. Of note, none of these studies documented a calculation for sample size.

### Stratified meta-analysis: Functional neurologic outcome

#### Sensorimotor

**Fig 2:**
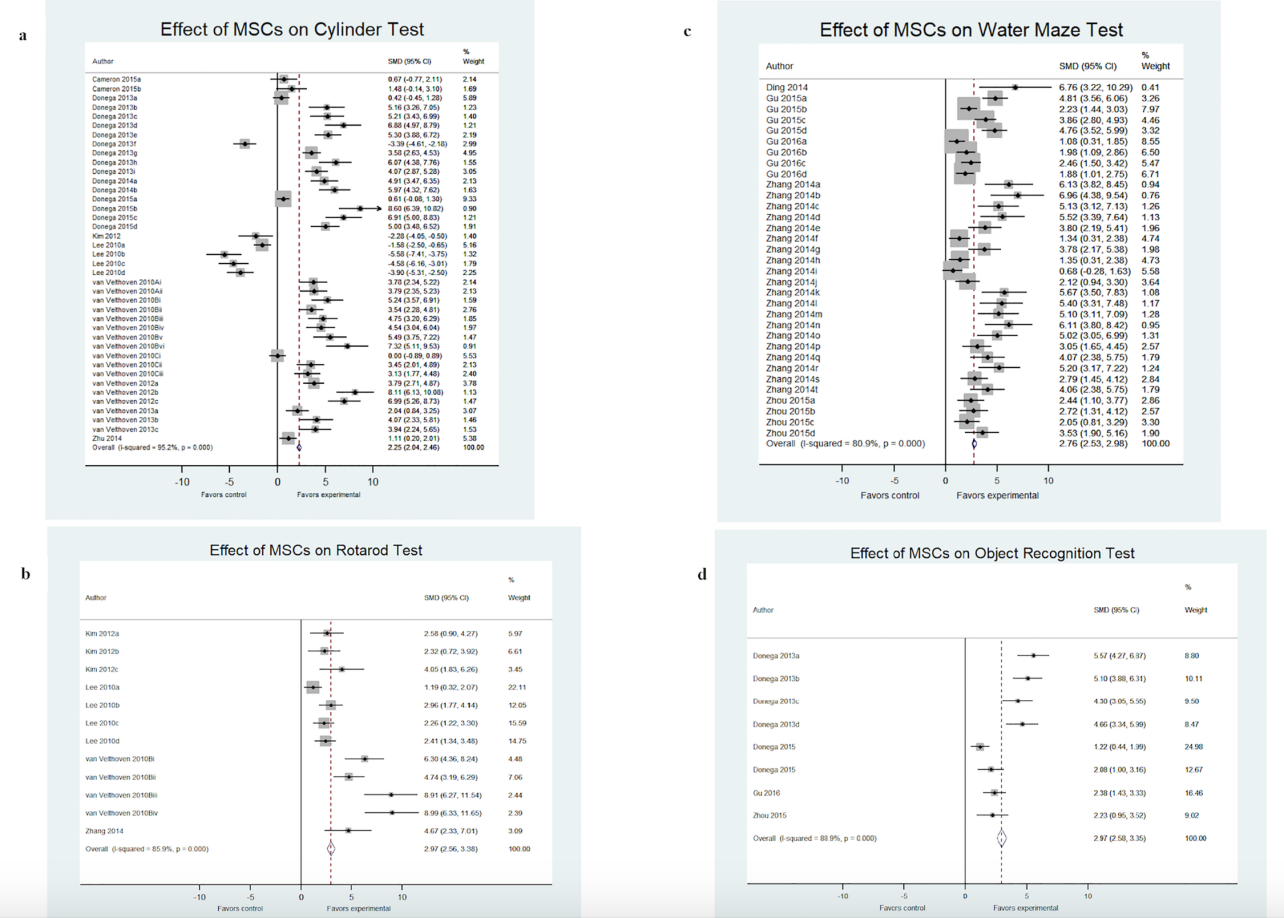
Effect size of MSCs across functional neurologic assessments from included studies. Forest plots demonstrating SMD and 95% CI for (a) cylinder rearing test, (b) rotarod test, (c) water maze test, and (d) novel object/object in place test.

Sensorimotor outcomes were assessed under two points: (1) cylinder rearing test and (2) rotarod test. Overall performance on the cylinder rearing test was improved by 2.25 SMD (95% CI, 2.04–2.46; 12 studies and 40 comparisons; Fig 2A). However, the heterogeneity between groups was significant (I^2^ = 95.2%; *p*<0.001). Six out of 14 interventions had effect sizes greater than 5.0 SMD, with 8.11 as the largest. Rotarod test performance was improved by 2.97 SMD (95% CI, 2.56–3.38; 4 studies and 12 comparisons; Fig 2B) with significant heterogeneity (I^2^ = 85.9%; *p*<0.001). Half of the studies resulted in an SMD greater than 3.0, with the largest effect size of 8.99.

Stratification by animal model, study design, and intervention characteristics revealed significant differences in effect size, seen in Table 2. For instance, groups exposed to hypoxic-ischemic insult on postnatal day seven or later had improved sensorimotor performance (2.92; 95% CI; 2.70–3.13). Effect size was larger in studies that used mice (3.19; 95% CI, 2.94–3.44), a carotid artery occlusion model of HIE (2.41; 95% CI 2.21–2.60), and isoflurane as the chosen anesthetic agent (2.92; 95% CI 2.70–3.13). Effect size was maximum when MSCs were allogeneic (2.81; 95% CI, 2.60–3.04), bone marrow-derived (2.39; 95% CI, 2.16–2.62), delivered intravenously (4.67; 95% CI, 2.33–7.01), and administered >72 hours after induction (2.01; 95% CI, 1.71–2.33) in multiple doses (4.22; 95% CI, 3.81–4.63).

**Table 2:** Stratification of estimated effect size for sensorimotor function.

Variable	# Studies	# Comparisons	SMD (95% CI)	% Weight	I^2^	p*	p**
**Animal model**							
**Species**							
Rat	7	25	0.99 (0.72–1.27)	44.62	94.5%	0.000	0.002
Mouse	7	29	3.19 (2.94–3.44)	55.38	93.6%	0.000	
**Strain**							
Sprague-Dawley	6	16	0.57 (0.25–0.89)	32.70	93.4%	0.000	0.000
C57/Bl6	8	38	3.01 (2.78–3.23)	67.30	94.1%	0.000	
**Gender**							
Male	3	14	0.40 (0.05–0.75)	27.99	93.9%	0.000	0.000
Mixed	3	8	3.09 (2.60–3.58)	13.96	92.5%	0.000	
Not reported	8	32	2.87 (2.62–3.11)	58.05	94.4%	0.000	
**Age (at HIE)**							
< 7 days	1	1	1.11 (0.20–2.01)	4.09	0.0%	0.000	0.001
7 days	3	11	0.31 (-0.06–0.68)	24.35	94.9%	0.000	
> 7 days	10	42	2.92 (2.70–3.13)	71.56	94.9%	0.000	
**Study Design**							
**HIE model**							
CAO + hypoxia	11	45	2.41 (2.21–2.60)	87.08	95.0%	0.000	0.081
MCAO	3	9	0.87 (0.36–1.38)	12.92	92.4%	0.000	
**Anesthetic**							
Isoflurane	10	42	2.92 (2.70–3.13)	71.56	93.9%	0.000	0.000
Halothane	1	2	1.03 (-0.05–2.10)	2.91	0.0%	0.463	
Ether	2	2	1.57 (0.73–2.42)	4.71	87.1%	0.000	
Ketamine	1	8	0.83 (-0.32–0.49)	20.82	96.1%	0.000	
**Intervention**							
**Source**							
Bone marrow	8	34	2.39 (2.16–2.62)	63.77	95.1%	0.000	0.420
Umbilical cord	4	6	1.55 (0.94–2.16)	8.96	84.8%	0.000	
Not reported	2	14	2.00 (1.65–2.35)	27.27	95.7%	0.000	
**Origin**							
Allogeneic	8	38	2.81 (2.60–3.04)	67.35	94.1%	0.000	0.003
Xenogeneic	6	16	0.95 (0.63–1.27)	32.65	94.9%	0.000	
**Dose**							
≤250,000	5	20	3.76 (3.41–4.10)	28.11	90.3%	0.000	0.002
>250,000 - ≤500,000	4	15	2.55 (2.23–2.88)	31.48	95.1%	0.000	
>500,000 - ≤1 million	4	18	0.70 (0.41–0.99)	39.17	94.7%	0.000	
>1 million	1	1	5.97 (4.32–7.62)	1.24	0.0%	0.000	
**Delivery**							
Intracerebral	5	19	4.39 (4.01–4.77)	23.63	85.9%	0.000	0.001
Intranasal	5	23	2.19 (1.93–2.46)	47.93	95.1%	0.000	
Intraperitoneal	1	1	1.11 (0.20–2.01)	4.09	0.0%	0.000	
Intracardiac	1	8	0.83 (-0.32–0.49)	20.81	96.1%	0.000	
Subcutaneous	1	2	1.03 (-0.05–2.10)	2.92	0.0%	0.463	
IV	1	1	4.67 (2.33–7.01)	0.62	0.0%	0.000	
**Timing**							
< 72 hrs	7	25	1.45 (1.18–1.73)	44.66	94.7%	0.000	0.003
> 72 hrs	6	16	2.01 (1.71–2.33)	35.16	95.4%	0.000	
Multiple doses	5	13	4.22 (3.81–4.63)	20.18	88.7%	0.000	
**Expansion media**							
DMEM	8	34	1.93 (1.70–2.16)	63.51	94.9%	0.000	0.400
HPL	1	2	5.37 (4.28–6.45)	2.86	0.0%	0.341	
Commercial	1	1	4.67 (2.33–7.01)	0.61	0.0%	0.000	
Not reported	4	17	2.43 (2.11–2.74)	33.02	4.9%	0.000	

Note

*p value for subgroup differences.

**p value for heterogeneity between subgroups with meta-regression analysis.

#### Cognitive

Cognitive outcomes were assessed under two points: (1) water maze and (2) object recognition (combines NORT and object in place test). Water maze performance was improved by 2.76 SMD (95% CI, 2.53–2.98; 5 studies and 33 comparisons; Fig 2C) with significant heterogeneity (I^2^ = 80.9%; *p*<0.001). Twenty out of 33 interventions were greater than 3.0 SMD, with the largest effect size of 6.96. Similarly, performance on variations of the object recognition test improved by 2.97 SMD (95% CI, 2.58–3.35; 4 studies and 8 comparisons; Fig 2D). Heterogeneity was again significant across these studies (I^2^ = 88.9%; *p*<0.001). In this comparison, four out of eight interventions were greater than 3.0 SMD.

Stratification by the aforementioned characteristics was performed for the cognitive results as well, seen in Table 3. Effect size was largest in studies that used mice (3.19; 95% CI, 2.74–3.63), a carotid artery occlusion model of HIE (2.66; 95% CI, 2.46–2.85), and chloral hydrate as the chosen anesthetic agent (6.76; 95% CI, 3.22–10.29). Intervention was most effective when MSCs were allogeneic (2.73, 95% CI, 2.47–2.98), placenta-derived (6.76; 95% CI, 3.22–10.29), delivered intraperitoneally (3.69; 95% CI 3.05–4.33), and administered <72 hours after induction (3.08, 95% CI, 2.81–3.36) in multiple doses (4.39; 95% CI, 3.61–5.18).

**Table 3:** Stratification of estimated effect size for cognitive function.

Variable	# Studies	# Comparisons	SMD (95% CI)	% Weight	I^2^	p*	p**
**Animal model**							
**Species**							
Rat	9	40	2.53 (2.33–2.73)	82.89	84.0%	0.000	0.637
Mouse	2	6	3.19 (2.74–3.63)	17.11	91.6%	0.000	
**Strain**							
Sprague-Dawley	7	35	2.34 (2.12–2.56)	69.84	83.3%	0.000	0.253
C57/Bl6	2	6	3.12 (2.74–3.63)	17.11	91.6%	0.000	
Wistar	2	5	3.57 (3.06–4.08)	13.05	81.5%	0.000	
**Gender**							
Male	7	35	2.34 (2.12–2.60)	69.84	83.3%	0.000	0.000
Mixed	2	6	3.19 (2.74–3.63)	17.11	91.6%	0.000	
Not reported	2	5	3.57 (3.06–4.08)	13.05	81.5%	0.000	
**Age (at HIE)**							
< 7 days	1	1	4.17 (2.63–5.71)	1.44	0.0%	0.000	0.396
7 days	6	34	2.50 (2.29–2.72)	72.24	86.1%	0.000	
> 7 days	2	6	3.19 (2.74–3.63)	17.11	91.6%	0.000	
Not reported	2	5	2.50 (1.89–3.11)	9.21	0.0%	0.682	
**Study Design**							
**HIE model**							
CAO + hypoxia	9	41	2.66 (2.46–2.85)	90.79	87.0%	0.000	0.264
Not reported	2	5	2.50 (1.89–3.11)	9.21	0.0%	0.682	
**Anesthetic**							
Isoflurane	2	6	3.19 (2.74–3.63)	17.11	91.6%	0.000	0.658
Halothane	3	6	1.64 (1.15–2.13)	14.30	79.1%	0.000	
Ether	2	19	2.94 (2.62–3.26)	33.47	85.5%	0.000	
Chloral	1	1	6.76 (3.22–10.29)	0.27	0.0%	0.000	
Not reported	3	9	2.47 (1.26–2.78)	34.85	83.7%	0.000	
**Intervention**							
**Source**							
Bone marrow	2	6	2.70 (2.30–3.09)	21.42	88.4%	0.000	0.961
Umbilical cord	5	30	2.55 (2.29–2.82)	47.76	84.7%	0.000	
Placenta	1	1	6.76 (3.22–10.29)	0.27	0.0%	0.000	
Not reported	3	9	2.71 (2.38–3.05)	30.55	88.9%	0.000	
**Origin**							
Allogeneic	6	16	2.73 (2.47–2.98)	52.24	87.5%	0.000	0.670
Xenogeneic	5	30	2.55 (2.29–2.82)	47.76	84.7%	0.000	
**Dose**							
≤250,000	5	11	1.78 (1.48–2.90)	36.38	0.0%	0.001	0.054
>250,000 - ≤500,000	3	25	2.92 (2.64–3.20)	42.54	88.1%	0.000	
>500,000 - ≤1 million	1	1	4.17 (2.63–5.71)	1.44	0.0%	0.003	
>1 million	2	9	3.53 (3.11–3.95)	19.64	67.8%	0.000	
**Delivery**							
Intracerebral	6	16	2.54 (1.99–2.52)	49.42	82.8%	0.000	0.047
Intranasal	2	6	3.19 (2.74–3.63)	17.11	91.6%	0.000	
Intraperitoneal	2	6	3.69 (3.05–4.33)	8.31	7.9%	0.366	
IV	1	18	2.69 (2.33–3.06)	25.16	87.3%	0.000	
**Timing**							
< 72 hrs	3	28	3.08 (2.81–3.36)	45.07	84.2%	0.000	0.158
> 72 hrs	6	15	2.05 (1.78–2.31)	49.36	84.6%	0.000	
Multiple doses	2	3	4.39 (3.61–5.18)	5.57	0.0%	0.877	
**Expansion media**							
DMEM	5	11	2.60 (2.26–2.95)	28.51	86.1%	0.000	0.716
Commercial	1	18	2.89 (2.56–3.21)	32.03	84.7%	0.000	
Alpha	1	1	6.76 (3.22–10.29)	0.27	0.0%	0.000	
Not reported	4	11	2.45 (2.15–2.74)	39.19	88.2%	0.000	

Note

*p value for subgroup differences.

**p value for heterogeneity between subgroups with meta-regression analysis.

#### Overall efficacy

When all studies and comparisons were combined, overall functional neurologic outcome improved by 2.42 SMD (95% CI, 2.29–2.56; 18 studies and 100 comparisons). More than 50% of these comparisons had effect sizes greater than 3.0 SMD. Heterogeneity remained high, which was expected when combining all comparisons and assessing variation in SMD (I^2^ = 92.6%; *p*<0.001).

### Narrative findings

Only one study investigated the effects of MSCs on staircase testing following HIE [30]. Outcomes were highly variable, making it difficult to include this measure in the meta-analysis. It is important however to mention that this study found a trend towards improvement in number of pellets eaten and lowest stair level reached by the MSC-treated group. Though not significant, the contralateral forelimb performance in the treated cohort was similar to that of the uninjured control group, suggesting a potential therapeutic effect.

MSCs are known to be neuroprotective against white matter injury. Three of the included studies assessed electrical findings in comparison groups. These results were not included in our meta-analysis given the limited number of studies that investigated electrical changes. Jellema *et al*. documented a significantly reduced number of electrographic seizures in the MSC treatment group following global HIE [31]. Two additional studies measured fEPSPs in hippocampal slices as a way of assessing long-term potentiation (LTP) recording. Zhou *et al*. found that LTP was significantly increased in the MSC-treated group, based on incremental mean slope of fEPSPs in response to high frequency stimulation [32]. Similarly, Gu *et al*. noted a trend towards increased LTP following MSC therapy. Together, these results support the idea that MSCs may be protective against the electrical consequences of HIE.

### Meta-regression analysis

Meta-regression was performed to simultaneously examine the impact of all variables on study effect. To further investigate the unaccounted heterogeneity across these studies, meta-regression and subgroup analysis were performed by animal model, study design, and intervention. For sensorimotor function, animal species/strain, gender, age at HIE induction, anesthetic, MSC origin, MSC dose, route of delivery, and timing of intervention were the significant sources of heterogeneity (*p*<0.05) (Table 2). For cognitive function, only gender and route of delivery were significant sources of heterogeneity (*p*<0.05) (Table 3).

### Publication bias

**Fig 3:**
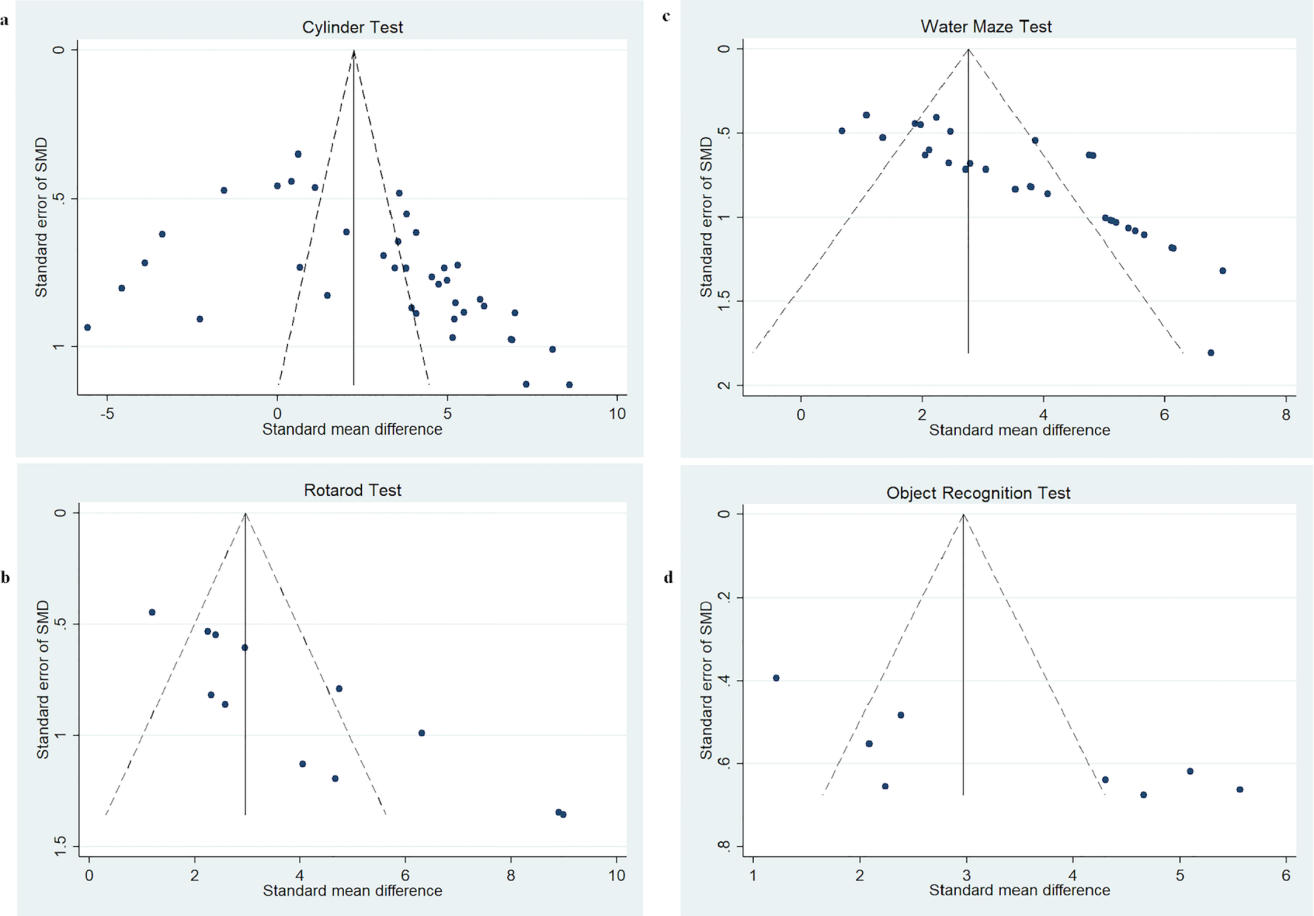
Funnel plots demonstrating publication bias from included studies. Funnel plots for (a) cylinder rearing test, (b) rotarod test, (c) water maze test, and (d) novel object/object in place test.

Funnel plots were created to examine the effect of study qualities and heterogeneity on publication bias (Fig 3). Asymmetry was detected in all funnel plots of sensorimotor and cognitive function, indicating the presence of publication bias in these studies. Egger’s tests were performed to formally detect statistical asymmetry, with a null hypothesis denying the existence of small study effects. The *p* value was <0.05 for all tests, indicating strong evidence to reject the null hypothesis in favor of the alternative (i.e. small study effect does exist).

## Discussion

Systematic reviews play a critical role in applying preclinical data to clinical practice. When combined with meta-analyses of these experiments, results can be assessed in a more methodical and objective manner. In preclinical and clinical studies of acute stroke, MSCs have shown to improve angiogenesis, neurogenesis, and functional outcomes in experimental treatment groups [33,34]. Investigators using animal models of HIE have demonstrated similarly promising results for this significant perinatal disorder. Progress in regenerative medicine has led to the first prospective randomized controlled clinical trial to evaluate the safety of similar treatments [25]. However, to our knowledge this is the first attempt to systematically collect and evaluate the current preclinical evidence supporting the use of MSCs in animal models of neonatal HIE. Based on the results of our meta-analysis, we conclude that there is indeed therapeutic potential of MSCs for improving functional neurologic outcome in affected neonates. Further, these results are applicable across a range of experimental conditions.

MSCs significantly improved overall neurobehavioral outcome in both sensorimotor and cognitive testing. Our pooled effect size of 2.42 is consistent with studies involving adult animal models of neurologic injury. For instance, a meta-analysis done by Peng *et al*. found an absolute effect size of 1.86 on overall sensorimotor function after traumatic brain injury [35]. In our study, the cylinder rearing results yielded an SMD of 2.25, which is comparable to the values obtained by Vahidy *et al* [36]. Furthermore, our rotarod estimates echoed the findings by Chen and colleagues, who examined the efficacy of neural stem cell use after ischemic stroke [37]. Taken collectively, these findings support the potential use of stem cell therapy in preclinical studies of neurologic disease.

Our meta-analysis suggests that further studies should be performed to elucidate the ideal MSC dose, as one study suggested quantities between 500,000 and 1,000,000 MSCs while another showed optimal doses over 1,000,000. Likewise, there was discrepancy regarding the most effective route of delivery, with sensorimotor results favoring an intravenous route while the cognitive studies favored intraperitoneal injection. These variables, amongst others, are exceedingly clinically relevant to future patient applications. As such, our findings should be used to guide future studies when determining the optimal MSC characteristics for successful outcomes.

In all comparisons, significant heterogeneity in treatment effect was found between study groups. This level of heterogeneity can be expected in studies such as ours based on the limited number of included studies and potential for bias in study selection. Funnel plots and Egger’s test for small-study effects confirmed the presence of publication bias. Study quality may also be affected if functional neurologic outcome, our primary outcome measure, was not the focus of the preclinical study. Such variations in study design could account for the heterogeneity found in our analysis.

We performed a meta-regression analysis to assess the effect of these variables and consider sources of heterogeneity. The results of this analysis suggest that sensorimotor and cognitive outcome can indeed be associated with moderator variables in the study. However, it is important to consider the limitations of meta-regression. In our analysis, there are relatively few studies, but many possible study characteristics that could explain heterogeneity. Without significant power, it is possible to arrive at false positive conclusions. Meta-regression is intended to generate hypotheses regarding heterogeneity, rather than explain them fully. For this reason, it is difficult to truly ascertain the variables with the most promising effects given the current collection of studies.

Use of the SYRCLE Risk of Bias tool highlighted notable deficiencies in reporting across all studies. None of the 19 studies included in our review were considered low risk of bias based on the reporting domains included in this tool. As discussed, domains were only scored as a low risk of bias if the authors specifically stated these details in their published manuscript. Therefore, it is possible that the studies utilized such methods in their trials but simply failed to report them. Our review emphasizes this widespread shortcoming and suggests a need for higher reporting standards when publishing, specifically for preclinical translational studies. We suggest using a checklist such as the SYRCLE Risk of Bias tool when designing future preclinical studies to minimize internal reporting bias.

Similarly, it is imperative that studies report all characteristics of their animal, experimental, and intervention models. For instance, one of the studies did not describe their specific method of hypoxic-ischemic insult or the age at which HIE was induced. Four studies did not report any of the established MSC characterization criteria. Additional studies were lacking at least one of these criteria. This information, along with information regarding the experimental conditions, is especially important for future study comparisons and meta-analyses. Without knowledge of these details, it becomes challenging when attempting to optimize interventions for future translational studies.

Our study has many strengths, primarily in that we conducted a systematic literature search and followed a published protocol method to ensure a diligent and rigorous review process. Data from multiple studies was combined in the meta-analysis, thus increasing the sample size and precision when studying effects of interest. Furthermore, our primary outcome of functional neurologic result is widely applicable to future preclinical and clinical studies.

On the other hand, our study has several limitations that are common across systematic reviews. For instance, included studies are limited to only those that have already been published. Unpublished data may exist that would further skew our results. While we made every effort to thoroughly search the current literature, it is possible that we may have missed relevant studies. Additionally, our meta-analysis is limited by a relatively small data set due to strict inclusion criteria, with external publication bias across these studies. Our study did not include experiments that used modified stem cells or those augmented by additional therapies such as hypothermia or granulocyte colony-stimulating factor. While the addition of these therapies has not demonstrated significant results in the aged brain, they may enhance the neurorestorative effect of MSCs in a more pliable neonatal brain [15,38]. Further, many of the included studies were primarily performed to investigate the histological or structural effects of MSC intervention on neonatal models of HIE, with functional neurologic outcome as a secondary measure. It is possible that these variations in study design have altered the results of our meta-analysis. Finally, we are unable to comment on the clinical safety of MSC therapy, as none of the included studies thoroughly investigated long-term effects on animal subjects. While immunogenicity is less of a concern with MSC therapy, other significant risks exist. For instance, MSCs have been associated with malignant transformation, tumor growth, and a higher overall degree of metastasis [39–41]. Although complications have been observed in humans receiving MSCs, meta-analyses have not shown a direct correlation between MSCs and acute toxicity, systemic failure, malignancy, or death [42–44]. The only noteworthy association is between MSCs and transient fever [45]. The long-term consequences of these effects are of particular concern in the developing preterm neonate. In future preclinical studies, animal subjects undergoing stem cell therapy for neonatal conditions should be followed into adulthood to determine the incidence of these and other adverse effects. Upcoming investigations should also examine the safety profile of cell-based products (conditioned media, exosomes, microRNA, *etc*.). Despite these limitations, our results reflect the widespread tendencies in this field of research.

Furthermore, this project limited the inclusion of experiments to those focusing on the neonatal brain. MSCs derived from both bone marrow and umbilical cord blood have repeatedly demonstrated the ability to restore neurogenesis and functional outcomes in young (2–3 months) and old rats (12–20 months) exposed to cerebral ischemia [33,34,46]. However, investigators have found that the age of the rat at the time of cerebral injury may influence the regenerative/reparative ability of the cell-based products. For instance, the success is limited in animal subjects with age-related comorbidities such as diabetes and hyperlipidemia [46]. These findings highlight the differences between the neuronal infrastructure and adaptation to injury in the neonatal versus mature brain. Future studies should be preformed in both adult and neonatal subjects under common comorbidities to determine if MSC outcomes are applicable throughout life.

## Conclusion

In brief, our findings suggest promising therapeutic potential for MSCs in the treatment of neonatal HIE. This meta-analysis of current preclinical studies demonstrates that MSC administration positively affects the functional neurologic status in treatment groups, as demonstrated by sensorimotor and cognitive testing. Results are amplified based on various animal model, study design, and intervention characteristics. However, further analysis is needed to determine the optimal MSC source, dose, timing, and route of administration. While study reporting, heterogeneity, and publication bias served as limitations to our review, the overall results are consistent with the current data from the field. These items should be taken into consideration when designing future preclinical studies and clinical trials.

## Supporting information

S1 File List of included studies. (DOCX)

S1 Table SYRCLE 2014 protocol format. (DOCX)

S2 Table Literature search terms (i.e. those used in PubMed). (DOCX)

S3 Table Detailed summary of information extracted from included studies. (DOCX)

S4 Table Neurobehavioral assessments used in included studies. (DOCX)

S5 Table MSC criteria reported by included studies. (DOCX)

S6 Table SYRCLE risk of bias assessment for included studies. (DOCX)

S1 Fig Bubble plot of sensorimotor outcome variables with fitted meta-regression line of standardized mean difference among. (a) animal, (b) strain, (c) gender, (d) age at HIE induction, (e) MSC source, (f) MSC origin, (g) MSC dose, (h) HIE model, (i) route of administration, (j) timing of administration, (k) anesthetic agents, (l) MSC expansion media.
(TIFF)

S2 Fig Bubble plot of cognitive outcome variables with fitted meta-regression line of standardized mean difference among. (a) animal, (b) strain, (c) age at HIE induction, (d) MSC source, (e) MSC origin, (f) MSC dose, (g) HIE model, (h) route of administration, (i) timing of administration, (j) anesthetic agents, (k) MSC expansion media.
(PNG)
