# Supplementary material for: Therapeutic potential of mesenchymal stromal cells for hypoxic ischemic encephalopathy: A systematic review and meta-analysis of preclinical studies
Source: PLoS One. 2017 Dec 19;12(12):e0189895. doi: 10.1371/journal.pone.0189895 (PMC5736208; doi:10.1371/journal.pone.0189895)
Supplement: S1 File — (DOCX) [file pone.0189895.s001.docx]

1. Cameron, S. H., Alwakeel, A. J., Goddard, L., Hobbs, C. E., Gowing, E. K., Barnett, E. R., … Oorschot, D. E. (2015). Delayed Post-Treatment with Bone Marrow-Derived Mesenchymal Stem Cells is Neurorestorative of Striatal Medium-Spiny Projection Neurons and Improves Motor Function After Neonatal Rat Hypoxia-Ischemia. Molecular and Cellular Neuroscience, 68, 56–72. <https://doi.org/10.1016/j.mcn.2015.03.019>
2. Ding, H.-F., Zhang, H., Ding, H.-F., Li, D., Yi, X.-H., Gao, X.-Y., … Ju, X.-L. (2014). Therapeutic Effect of Placenta-Derived Mesenchymal Stem Cells on Hypoxic-Ischemic Brain Damage in Rats. World Journal of Pediatrics, 11(1), 74–82. <https://doi.org/10.1007/s12519-014-0531-8>
3. Donega, V., Nijboer, C. H., Braccioli, L., Slaper-Cortenbach, I., Kavelaars, A., Van Bel, F., & Heijnen, C. J. (2014). Intranasal Administration of Human MSC for Ischemic Brain Injury in the Mouse: In Vitro and In Vivo Neuroregenerative Nunctions. PLoS ONE, 9(11), e112339. <https://doi.org/10.1371/journal.pone.0112339>
4. Donega, V., Nijboer, C. H., van Velthoven, C. T., Youssef, S. a, de Bruin, A., van Bel, F., … Heijnen, C. J. (2015). Assessment of Long-Term Safety and Efficacy of Intranasal Mesenchymal Stem Cell Treatment for Neonatal Brain Injury in the Mouse. Pediatric Research, 78(5), 520–526. <https://doi.org/10.1038/pr.2015.145>
5. Donega, V., van Velthoven, C. T. J., Nijboer, C. H., van Bel, F., Kas, M. J. H., Kavelaars, A., & Heijnen, C. J. (2013). Intranasal Mesenchymal Stem Cell Treatment for Neonatal Brain Damage: Long-Term Cognitive and Sensorimotor Improvement. PLoS ONE, 8(1), e51253. <https://doi.org/10.1371/journal.pone.0051253>
6. Gu, Y., He, M., Zhou, X., Liu, J., Hou, N., Bin, T., … Chen, J. (2016). Endogenous IL-6 of mesenchymal stem cell improves behavioral outcome of hypoxic-ischemic brain damage neonatal rats by supressing apoptosis in astrocyte. Scientific Reports, 6(1), 18587. <https://doi.org/10.1038/srep18587>
7. Gu, Y., Zhang, Y., Bi, Y., Liu, J., Tan, B., Gong, M., … Chen, J. (2015). Mesenchymal stem cells suppress neuronal apoptosis and decrease IL-10 release via the TLR2/NF$κ$B pathway in rats with hypoxic-ischemic brain damage. Molecular Brain, 8(1), 65. <https://doi.org/10.1186/s13041-015-0157-3>
8. Jellema, R. K., Wolfs, T. G. A. M., Lima Passos, V., Zwanenburg, A., Ophelders, D. R. M. G., Kuypers, E., … Kramer, B. W. (2013). Mesenchymal Stem Cells Induce T-Cell Tolerance and Protect the Preterm Brain after Global Hypoxia-Ischemia. PLoS ONE, 8(8), 1–15. <https://doi.org/10.1371/journal.pone.0073031>
9. Kim, E. S., Ahn, S. Y., Im, G. H., Sung, D. K., Park, Y. R., Choi, S. H., … Park, W. S. (2012). Human umbilical cord blood–derived mesenchymal stem cell transplantation attenuates severe brain injury by permanent middle cerebral artery occlusion in newborn rats. Pediatric Research, 72(3), 277–284. <https://doi.org/10.1038/pr.2012.71>
10. Lee, J. A., Kim, B. Il, Jo, C. H., Choi, C. W., Kim, E. K., Kim, H. S., … Choi, J. H. (2010). Mesenchymal stem-cell transplantation for hypoxic-ischemic brain injury in neonatal rat model. Pediatric Research, 67(1), 42–46. <https://doi.org/10.1203/PDR.0b013e3181bf594b>
11. van Velthoven, C. T. J., Kavelaars, A., van Bel, F., & Heijnen, C. J. (2010). Mesenchymal stem cell treatment after neonatal hypoxic-ischemic brain injury improves behavioral outcome and induces neuronal and oligodendrocyte regeneration. Brain, Behavior, and Immunity, 24(3), 387–393. <https://doi.org/10.1016/j.bbi.2009.10.017>
12. van Velthoven, C. T. J., Kavelaars, A., van Bel, F., & Heijnen, C. J. (2010). Repeated Mesenchymal Stem Cell Treatment after Neonatal Hypoxia-Ischemia Has Distinct Effects on Formation and Maturation of New Neurons and Oligodendrocytes Leading to Restoration of Damage, Corticospinal Motor Tract Activity, and Sensorimotor Function. Journal of Neuroscience, 30(28), 9603–9611. <https://doi.org/10.1523/JNEUROSCI.1835-10.2010>
13. van Velthoven, C. T. J., Kavelaars, A., Van Bel, F., & Heijnen, C. J. (2010). Nasal administration of stem cells: A promising novel route to treat neonatal ischemic brain damage. Pediatric Research, 68(5), 419–422. <https://doi.org/10.1203/PDR.0b013e3181f1c289>
14. van Velthoven, C. T. J., Van De Looij, Y., Kavelaars, A., Zijlstra, J., Van Bel, F., Huppi, P. S., … Heijnen, C. J. (2012). Mesenchymal stem cells restore cortical rewiring after neonatal ischemia in mice. Annals of Neurology, 71(6), 785–796. <https://doi.org/10.1002/ana.23543>
15. van Velthoven, C. T., Sheldon, R. a, Kavelaars, A., Derugin, N., Vexler, Z. S., Willemen, H. L., … Ferriero, D. M. (2013). Mesenchymal stem cell transplantation attenuates brain injury after neonatal stroke. Stroke, 44(5), 1426–1432. <https://doi.org/10.1161/STROKEAHA.111.000326>
16. Xia, G., Hong, X., Chen, X., Lan, F., Zhang, G., & Liao, L. (2010). Intracerebral transplantation of mesenchymal stem cells derived from human umbilical cord blood alleviates hypoxic ischemic brain injury in rat neonates. Journal of Perinatal Medicine, 38(2), 215–221. <https://doi.org/10.1515/JPM.2010.021>
17. Zhang, X., Zhang, Q., Li, W., Nie, D., Chen, W., Xu, C., … Tu, W. (2014). Therapeutic effect of human umbilical cord mesenchymal stem cells on neonatal rat hypoxic-ischemic encephalopathy. Journal of Neuroscience Research, 92(1), 35–45. <https://doi.org/10.1002/jnr.23304>
18. Zhou, X., Gu, J., Gu, Y., He, M., Bi, Y., Chen, J., & Li, T. (2015). Human umbilical cord-derived mesenchymal stem cells improve learning and memory function in hypoxic-ischemic brain-damaged rats via an IL-8-mediated secretion mechanism rather than differentiation pattern induction. Cellular Physiology and Biochemistry, 35(6), 2383–2401. <https://doi.org/10.1159/000374040>
19. Zhu, L. H., Bai, X., Zhang, N., Wang, S. Y., Li, W., & Jiang, L. (2014). Improvement of human umbilical cord mesenchymal stem cell transplantation on glial cell and behavioral function in a neonatal model of periventricular white matter damage. Brain Research, 1563, 13–21. <https://doi.org/10.1016/j.brainres.2014.03.030>
