# Supplementary material for: Therapeutic potential of mesenchymal stromal cells for hypoxic ischemic encephalopathy: A systematic review and meta-analysis of preclinical studies
Source: PLoS One. 2017 Dec 19;12(12):e0189895. doi: 10.1371/journal.pone.0189895 (PMC5736208; doi:10.1371/journal.pone.0189895)
Supplement: S1 Table — (DOCX) [file pone.0189895.s002.docx]

| **Supplementary Table 1.** SYRCLE 2014 Protocol Format  **Systematic Review Protocol for Animal Intervention Studies**  **Format by SYRCLE (**[**www.syrcle.nl**](http://www.syrcle.nl)**)**  **Version 2.0 (December 2014)** | | | | |
| --- | --- | --- | --- | --- |
| **Item #** | **Section/Subsection/Item** | **Description** | | **Check for included** |
|  | A. General | | | |
| 1. | Title of the review | Therapeutic Potential of Mesenchymal Stromal Cells for Hypoxic Ischemic Encephalopathy: A Systematic Review and Meta-Analysis of Preclinical Studies | | X |
| 2. | Authors (names, affiliations, contributions) | **Jamie Archambault^1^:** conception and study design, data collection and analysis, manuscript drafting  **Dawn McDaniel^1^:** conception and study design  **Lauryn Winter^1^:** manuscript revision  **Peter Hornsby^2^:** manuscript revision, scientific input from the field  **Alvaro Moreira^1^:** conception and study design, data collection and analysis, manuscript drafting, supervision  ^1^Department of Pediatrics, Division of Neonatology, UT Health San Antonio  ^2^Department of Cellular and Integrative Physiology, Barshop Institute for Longevity and Aging Studies, University of Texas Health Science Center at San Antonio | | X |
| 3. | Other contributors (names, affiliations, contributions) | None | | X |
| 4. | Contact person + e-mail address | **Jamie Archambault:** [archambault@livemail.uthscsa.edu](mailto:archambault@livemail.uthscsa.edu)  **Alvaro Moreira:** [moreiraa@uthscsa.edu](mailto:moreiraa@uthscsa.edu) | | X |
| 5. | Funding sources/sponsors | National Center for Advancing Translational Sciences, National Institutes of Health, through Grant **KL2 TR001118.** The content is solely the responsibility of the authors and does not necessarily represent the official views of the NIH. | | X |
| 6. | Conflicts of interest | None | | X |
| 7. | Date and location of protocol registration | January 23, 2017  CAMARADES | | X |
| 8. | Registration number (if applicable) | Not applicable | | -- |
| 9. | Stage of review at time of registration | Preliminary searches  Piloting study selection  Formal screening with final search criteria | | X |
|  | B. Objectives | | | |
|  | Background | | | |
| 10. | What is already known about this disease/model/intervention? Why is it important to do this review? | Hypoxic ischemic encephalopathy (HIE) is a devastating neurologic condition with high mortality rates and significant long-term complications for infants who survive. Mesenchymal stromal cells (MSCs) have emerged as novel therapeutic agents shown to have promising results in experimental studies of HIE.  The purpose of this systematic review is to assess the efficacy of exogenous administration of MSCs in animal models of HIE. | | X |
|  | Research question | | | |
| 11. | Specify the disease/health problem of interest | HIE | | X |
| 12. | Specify the population/species studied | Neonatal animal model of HIE | | X |
| 13. | Specify the intervention/exposure | Mesenchymal stromal cells | | X |
| 14. | Specify the control population | Placebo or no treatment | | X |
| 15. | Specify the outcome measures | Primary outcome: functional neurologic outcome  -Cognitive  -Motor  -Sensory  Secondary outcome: lesion size  -Neuroimaging  -Histologic brain damage | | X |
| 16. | State your research question (based on items 11-15) | What are the effects of MSCs on experimental HIE? | | X |
|  | C. Methods | | | |
|  | Search and study identification | | | |
| 17. | Identify literature databases to search (*e.g.* Pubmed, Embase, Web of science) | X MEDLINE via PubMed  X Web of Science  □SCOPUS  □EMBASE  X Other, namely: CINAHL, Google Scholar  □Specific journal(s), namely: | | X |
| 18. | Define electronic search strategies (*e.g.* use the [step by step search guide^15^](http://www.ncbi.nlm.nih.gov/pmc/articles/PMC3265183/pdf/LA-11-087.pdf) and animal search filters[^20,^](http://www.ncbi.nlm.nih.gov/pmc/articles/PMC3104815/pdf/LA-09-117.pdf) [^21^](http://lan.sagepub.com/content/48/1/88.full.pdf+html)) | When available, please add a supplementary file containing your search strategy: [Supplementary Table 2] | | X |
| 19. | Identify other sources for study identification | X Reference lists of included studies  X Reference lists of relevant reviews  □Books  □Conference proceedings, namely:  □Contacting authors/ organisations, namely:  □Other, namely: | | X |
| 20. | Define search strategy for these other sources | Screening the reference lists for relevant titles and screening the abstracts of these relevant titles | | X |
|  | Study selection | | | |
| 21. | Define screening phases (*e.g.* pre-screening based on title/abstract, full text screening, both) | First phase screening by title and abstract  Second phase full text screening of eligible articles | | X |
| 22. | Specify (a) the number of reviewers per screening phase and (b) how discrepancies will be resolved | a) Two investigators (J. Archambault and A. Moreira) will independently screen all the abstracts/full texts for the inclusion criteria. b) Differences of opinion in either phase that cannot be resolved by discussion will be resolved by consulting a third investigator (D. McDaniel). | | X |
|  | *Define all inclusion and exclusion criteria based on:* | | | |
| 23. | Type of study (design) | Inclusion criteria:  Animal intervention studies (with control group), regardless of the methodological quality  Exclusion criteria:  Non-intervention studies, no control group, co-intervention studies | | X |
| 24. | Type of animals/population (*e.g.* age, gender, disease model) | Inclusion criteria:  Healthy animals  Neonatal animal models of experimental HIE  All genders  Disease model: hypoxia and ischemia  Exclusion criteria:  Humans  Non-neonatal animal models of brain injury  In vitro | | X |
| 25. | Type of intervention (*e.g.* dosage, timing, frequency) | Inclusion criteria:  Administration of MSCs – all dosages, timing, and frequency  MSC as defined by International Society for Cellular Therapy  Exclusion criteria:  Modified MSCs  Progenitor cells  MSCs combined with other therapies  Acellular derivatives | | X |
| 26. | Outcome measures | Inclusion criteria:  Primary outcome: functional neurologic outcome  Secondary outcome: lesion size  Exclusion criteria:  Other outcome measures | | X |
| 27. | Language restrictions | Inclusion criteria:  English  Exclusion criteria:  All other languages | | X |
| 28. | Publication date restrictions | Inclusion criteria:  All publication dates  Exclusion criteria:  None | | X |
| 29. | Other | Inclusion criteria:  Not applicable  Exclusion criteria:  Not applicable | | X |
| 30. | Sort and prioritize your exclusion criteria per selection phase | Selection phase: title and abstract screening  1. Not a primary study  2. Not an in vivo animal study  3. Not HIE  4. No MSC treatment  5. Adult animal  Selection phase: full text screening  1. Not a primary study  2. Not an in vivo animal study  3. No HIE model  4. No MSC treatment  5. No control group  5. Co-intervention studies | | X |
|  | Study characteristics to be extracted (for assessment of external validity, reporting quality) | | | |
| 31. | Study ID (*e.g.* authors, year) | Authors, journal, title, year, language, contact author e-mail | | X |
| 32. | Study design characteristics (*e.g.* experimental groups, number of animals) | Number of animals in experimental and control groups  Method of HIE induction | | X |
| 33. | Animal model characteristics (*e.g.* species, gender, disease induction) | Animal species, strain, age, gender, and immune status | | X |
| 34. | Intervention characteristics (*e.g.* intervention, timing, duration) | Source, dose, delivery, timing, and frequency of MSCs | | X |
| 35. | Outcome measures | Type and timing of outcome measures in paper | | X |
| 36. | Other (*e.g.* drop-outs) | Reason of exclusion | | X |
|  | Assessment risk of bias (internal validity) or study quality | | | |
| 37. | Specify (a) the number of reviewers assessing the risk of bias/study quality in each study and (b) how discrepancies will be resolved | a) Two investigators (J. Archambault and A. Moreira) will independently assess the risk of bias/study quality in each study. b) Differences of opinion that cannot be resolved by discussion will be resolved by consulting a third investigator (D. McDaniel). | | X |
| 38. | Define criteria to assess (a) the internal validity of included studies (*e.g.* selection, performance, detection and attrition bias) and/or (b) other study quality measures (*e.g.* reporting quality, power) | X By use of [SYRCLE's Risk of Bias tool^4^](http://www.biomedcentral.com/1471-2288/14/43/abstract)  □ By use of SYRCLE’s Risk of Bias tool, adapted as follows:  □By use of [CAMARADES' study quality checklist, e.g ^22^](http://www.ncbi.nlm.nih.gov/pubmed/15060322)  □By use of CAMARADES' study quality checklist, adapted as follows:  □Other criteria, namely: | | X |
|  | Collection of outcome data | | | |
| 39. | For each outcome measure, define the type of data to be extracted (*e.g.* continuous/dichotomous, unit of measurement) | Primary outcome: functional neurologic outcome  -Cognitive: continuous  -Motor: continuous  -Sensory: continuous  Secondary outcome: lesion size  -Neuroimaging: continuous  -Histologic brain damage: continuous | | X |
| 40. | Methods for data extraction/retrieval (*e.g.* first extraction from graphs using a digital screen ruler, then contacting authors) | Extraction from text, tables, and figures (GetData graph digitizer 2.26)  Contact authors in case of missing data | | X |
| 41. | Specify (a) the number of reviewers extracting data and (b) how discrepancies will be resolved | a) Two investigators (J. Archambault and A. Moreira) will independently extract data from the included studies. b) Differences of opinion that cannot be resolved by discussion will be resolved by consulting a third investigator (D. McDaniel). | | X |
|  | Data analysis/synthesis | | | |
| 42. | Specify (per outcome measure) how you are planning to combine/compare the data (*e.g.* descriptive summary, meta-analysis) | For sufficient data, we will conduct a meta-analysis for eligible studies. If insufficient data to measure outcomes, we will provide a descriptive summary of study results. | | X |
| 43. | Specify (per outcome measure) how it will be decided whether a meta-analysis will be performed | High heterogeneity is expected between studies due to differences in the study designs. We will preform a meta-regression analysis to investigate sources of heterogeneity. | | X |
|  | *If a meta-analysis seems feasible/sensible, specify (for each outcome measure):* | | | |
| 44. | The effect measure to be used (*e.g.* mean difference, standardized mean difference, risk ratio, odds ratio) | Continuous outcomes will be analysed using standardized mean differences (95% CI) | | X |
| 45. | The statistical model of analysis (*e.g.* random or fixed effects model) | Random effects model. | | X |
| 46. | The statistical methods to assess heterogeneity (*e.g.* I^2^, Q) | I^2^ | | X |
| 47. | Which study characteristics will be examined as potential source of heterogeneity (subgroup analysis) | Study design: experimental and control groups, HIE model, anesthetic  Animal model: species, strain, age, gender, immune status  MSC intervention: source, dose, delivery, timing, frequency | | X |
| 48. | Any sensitivity analyses you propose to perform | Not applicable | | -- |
| 49. | Other details meta-analysis (*e.g.* correction for multiple testing, correction for multiple use of control group) | Not applicable | | -- |
| 50. | The method for assessment of publication bias | Funnel plot assessment  Egger’s regression | | X |
|  | | | | |
| Final approval by (names, affiliations): | | Jamie Archambault; Alvaro Moreira  UT Health San Antonio  7703 Floyd Curl Drive MC 7812  San Antonio, TX, USA 78229 |  | |
