# Supplementary material for: Therapeutic potential of mesenchymal stromal cells for hypoxic ischemic encephalopathy: A systematic review and meta-analysis of preclinical studies
Source: PLoS One. 2017 Dec 19;12(12):e0189895. doi: 10.1371/journal.pone.0189895 (PMC5736208; doi:10.1371/journal.pone.0189895)
Supplement: S2 Table — (DOCX) [file pone.0189895.s003.docx]

**Supplementary Table 2.** Literature search terms (i.e. those used in PubMed)

(((((((("hypoxia-ischemia, brain"[MeSH Terms] OR ("hypoxic ischaemic encephalopathy"[All Fields] OR "hypoxia-ischemia, brain"[MeSH Terms] OR ("hypoxia-ischemia"[All Fields] AND "brain"[All Fields]) OR "brain hypoxia-ischemia"[All Fields] OR ("hypoxic"[All Fields] AND "ischemic"[All Fields] AND "encephalopathy"[All Fields]) OR "hypoxic ischemic encephalopathy"[All Fields])) OR HIE[All Fields]) OR ("ischaemia"[All Fields] OR "ischemia"[MeSH Terms] OR "ischemia"[All Fields])) OR ("asphyxia"[MeSH Terms] OR "asphyxia"[All Fields])) AND ((("cerebrum"[MeSH Terms] OR "brain"[MeSH Terms]) OR ("cerebrum"[MeSH Terms] OR "cerebrum"[All Fields] OR "cerebral"[All Fields] OR "brain"[MeSH Terms] OR "brain"[All Fields])) OR ("brain"[MeSH Terms] OR "brain"[All Fields]))) AND (("mesenchymal stromal cells"[MeSH Terms] OR ("mesenchymal stromal cells"[MeSH Terms] OR ("mesenchymal"[All Fields] AND "stromal"[All Fields] AND "cells"[All Fields]) OR "mesenchymal stromal cells"[All Fields] OR ("mesenchymal"[All Fields] AND "stromal"[All Fields] AND "cell"[All Fields]) OR "mesenchymal stromal cell"[All Fields])) OR ("mesenchymal stromal cells"[MeSH Terms] OR ("mesenchymal"[All Fields] AND "stromal"[All Fields] AND "cells"[All Fields]) OR "mesenchymal stromal cells"[All Fields] OR ("mesenchymal"[All Fields] AND "stem"[All Fields] AND "cell"[All Fields]) OR "mesenchymal stem cell"[All Fields]))) AND (((("neonatology"[MeSH Terms] OR ("neonatology"[MeSH Terms] OR "neonatology"[All Fields])) OR ("infant, newborn"[MeSH Terms] OR ("infant"[All Fields] AND "newborn"[All Fields]) OR "newborn infant"[All Fields] OR "neonate"[All Fields])) OR ("infant"[MeSH Terms] OR "infant"[All Fields])) OR ("premature birth"[MeSH Terms] OR ("premature"[All Fields] AND "birth"[All Fields]) OR "premature birth"[All Fields] OR "premature"[All Fields]))) AND (preclinical[All Fields] OR ("animals"[MeSH Terms:noexp] OR animal[All Fields]))) NOT ("review"[Publication Type] OR "review literature as topic"[MeSH Terms] OR "review"[All Fields])
