# Supplementary material for: Therapeutic potential of mesenchymal stromal cells for hypoxic ischemic encephalopathy: A systematic review and meta-analysis of preclinical studies
Source: PLoS One. 2017 Dec 19;12(12):e0189895. doi: 10.1371/journal.pone.0189895 (PMC5736208; doi:10.1371/journal.pone.0189895)
Supplement: S3 Table — (DOCX) [file pone.0189895.s004.docx]

**Supplementary Table 3.**  Detailed summary of information extracted from included studies

| **Author (year)** | **Study Design** | | **Animal Characteristics** | | | **Intervention Characteristics** | | **Primary Outcome** | |
| --- | --- | --- | --- | --- | --- | --- | --- | --- | --- |
|  | **HIE Model** | **Anesthetic** | **Animal Model; Gender** | **Age** | **Immuno-deficient?** | **Source; (Origin)** | **Dose; Delivery;  Timing; Frequency** | **Cognitive** | **Sensori- motor** |
| Cameron (2015) | Right CAL, followed by 90 min of hypoxia (8% O2) 2.5 hrs after ligation | Nitrous oxide + halothane | Sprague-Dawley rat;  male | HIE on PN7 | No | Bone marrow from adult male Sprague-Dawley rats (allogeneic) | 0.75-1 x 10^6^; subcutaneous;  7 days post-HIE; 1x |  | **CRT Staircase test** |
| Ding (2014) | Left CAL, followed by 2.5 hrs of hypoxia (8% O2) 2 hrs after ligation | Chloral hydrate | Wistar rat; not reported | HIE on PN7 | No | Term placenta from healthy rats (allogeneic) | 5 x 10^5^; intracerebral; 2 days post-HIE; 1x | **Water maze** |  |
| Donega (2013) | Right CAL, followed by 45 min of hypoxia (10% O2) immediately after ligation | Isoflurane | C57BL/6 mouse; not reported | HIE on PN9 | No | C56Bl/6 mice (allogeneic) | 0.25-1 x 10^6^; intranasal; 3, 10, 17, or 3+10  days post-HIE; 1x or 2x | **NORT** | **CRT** |
| Donega (2014) | Right CAL, followed by 45 min of hypoxia (10% O2) immediately after ligation | Isoflurane | C57BL/6 mouse; not reported | HIE on PN9 | No | Bone marrow from healthy human donors (xenogeneic) | 1-2 x 10^6^; intranasal; 10 days post-HIE; 1x |  | **CRT** |
| Donega (2015) | Right CAL, followed by 45 min of hypoxia (10% O2) immediately after ligation | Isoflurane | C57BL/6 mouse; male and female | HIE on PN9 | No | Bone marrow from C57BL/6 mice (allogeneic) | 0.5 x 10^6^; intranasal; 10 days post-HIE; 1x | **NORT** | **CRT** |
| Gu (2015) | Left CAL, followed by 2.5 hrs of hypoxia (8% O2) 2 hrs after ligation | Not reported | Wistar rat; not reported | HIE on PN7 | No | Bone marrow from 3-4 week old rats (allogeneic) | 1.5 x 10^6^; intracerebroventricular; 2 days post-HIE; 1x | **Water maze** |  |
| Gu (2016) | Left CAL, followed by 2.5 hrs of hypoxia (8% O2) 2 hrs after ligation | Not reported | Sprague-Dawley rat; not reported | HIE on PN7 | Yes | Rats (allogeneic) | 2 x 10^5^; intracerebroventricular; 5 days post-HIE; 1x | **Water maze NORT fEPSP** |  |
| Jellema (2013) | UCO via an inflatable vascular occluder for 25 min, followed by reperfusion for 7 days | Not reported | Texel sheep; not reported | HIE on  gestational day 105.5 ± 1.1 | No | Bone marrow from healthy human male donor (xenogeneic) | 3.5 x 10^6^; IV; 1 hour post-HIE; 1x | **EEG  seizure burden** |  |
| Kim (2012) | Right MCAO | Isoflurane + pentobarbitol | Sprague-Dawley rat; male | HIE on PN10 | No | Umbilical cord blood from human donors (xenogeneic) | 1 x 10^5^; intraventricular; 6 hours post-HIE; 1x |  | **CRT RR** |
| Lee (2010) | Bilateral CAL with severance of the left common carotid artery, followed by 3.5 hrs of hypoxia (8% O2) 2 hrs after ligation | Ketamine + xylazine | Sprague-Dawley rat; male | HIE on PN7 | No | Bone marrow from human donors undergoing orthopedic surgery (xenogeneic) | 1 x 10^6^; intracardiac; 3 days post-HIE; 1x |  | **CRT RR** |
| van Velthoven (2010)A | Right CAL, followed by 45 min of hypoxia (10% O2) immediately after ligation | Isoflurane | C57BL/6 mouse; male and female | HIE on PN9 | No | Bone marrow from femur and tibia of 6-8 wk old C57Bl/6-Tg (UBC-GFP) 30Dcha/J mice (allogeneic) | 1 x 10^5^; intracerebral; 3 or 10 day post-HIE; 1x |  | **CRT** |
| van Velthoven (2010)B | Right CAL followed by 45 min of hypoxia (10% O2) immediately after ligation | Isoflurane | C57BL/6 mouse; not reported | HIE on PN9 | No | Bone marrow from femur and tibia of 6-8 wk old C57Bl/6-Tg (UBC-GFP) 30Dcha/J mice (allogeneic) | 1 x 10^5^; intracerebral; 3 or 3+10 day post-HIE; 1x or 2x |  | **CRT RR** |
| van Velthoven (2010)C | Right CAL, followed by 45 min of hypoxia (10% O2) immediately after ligation | Isoflurane | C57BL/6 mouse; male and female | HIE on PN9 | No | Bone marrow from femur and tibia of 6-8 wk old C57Bl/6-Tg (UBC-GFP) 30Dcha/J mice (allogeneic) | 5 x 10^5^; intranasal s/p hyaluronidase; 10 days post-HIE; 1x |  | **CRT** |
| van Velthoven (2012) | Right CAL followed by 45 min of hypoxia (10% O2) immediately after ligation | Isoflurane | C57BL/6 mouse; not reported | HIE on PN9 | No | 8 wk old C57Bl/6-Tg (UBC-GFP) 30Dcha/J mice (allogeneic) | 1 x 10^5^; intracerebral; 3+10 days post-HIE; 2x |  | **CRT** |
| van Velthoven (2013) | Right MCAO | Isoflurane | Sprague-Dawley rat; not reported | HIE on PN10 | No | Sprague Dawley rat (allogeneic) | 1 x 10^6^; intranasal s/p hyaluronidase; 3 days post-HIE; 1x |  | **CRT Adhesive removal test** |
| Xia (2010) | Left CAL, followed by 2.5 hrs of hypoxia (8% O2) 3 hrs after ligation | Halothane | Sprague-Dawley rat; not reported | HIE on PN7 | Yes - daily intraperitoneal injection of cyclosporin A from 2 days post-HIE until death | Umbilical cord blood from full-term human newborns (xenogeneic) | 1 x 10^5^; intracerebroparenchymal; 3 days post-HIE; 1x |  | **mNSS** |
| Zhang (2014) | Right CAL, followed by 2 hrs of hypoxia (8% O2) 2-3 hrs after ligation | Ether | Sprague-Dawley rat; not reported | HIE on PN7 | No | Wharton's jelly from human umbilical cords (xenogeneic) | 5 x 10^5^ (IV)  or 5 x 10^6^ (IP); IV or intraperitoneal; 1 or 3 days post-HIE; 1x | **Water maze** | **RR Longa score** |
| Zhou (2015) | Unclear - Rice model | Not reported | Sprague-Dawley rat; not reported | Not reported | No | Human umbilical cord (xenogeneic) | 2 x 10^5^; intracerebroventricular; 5 days post-HIE; 1x | **Water maze NORT fEPSP** |  |
| Zhu (2014) | Left CAL, followed by 4 hrs of hypoxia (6% O2) 2 hrs after ligation | Ether | Sprague-Dawley rat; not reported | HIE on PN3 | No | Human umbilical cord (xenogeneic) | 1 x 10^6^; intraperitoneal; 1, 2, and 3 days post-HIE; 3x |  | **CRT Open field test** |
| **Abbreviations:** carotid artery ligation (CAL); middle cerebral artery occlusion (MCAO); hypoxic ischemic encephalopathy (HIE); postnatal (PN); intravenous (IV); intraperitoneal (IP); cylinder rearing test (CRT); rotarod (RR); novel object recognition test (NORT); field excitatory postsynaptic potential (fEPSP) | | | | | | | | | |
