# Supplementary material for: Therapeutic potential of mesenchymal stromal cells for hypoxic ischemic encephalopathy: A systematic review and meta-analysis of preclinical studies
Source: PLoS One. 2017 Dec 19;12(12):e0189895. doi: 10.1371/journal.pone.0189895 (PMC5736208; doi:10.1371/journal.pone.0189895)
Supplement: S4 Table — (DOCX) [file pone.0189895.s005.docx]

**Supplementary Table 4.** Neurobehavioral assessments used in included studies

| **Author (Year)** | **Motor** | | | | **Cognitive** | | | **Compiled Scores** | | **Electric** | |
| --- | --- | --- | --- | --- | --- | --- | --- | --- | --- | --- | --- |
|  | **Cylinder Rearing** | **Rotarod** | **Staircase** | **Adhesive Removal** | **Water Maze** | **Novel Object/**  **Object in Place** | **Open Field** | **mNSS** | **Longa Score** | **EEG Seizure Burden** | **fEPSP** |
| Cameron (2015) | X |  | X |  |  |  |  |  |  |  |  |
| Ding (2014) |  |  |  |  | X |  |  |  |  |  |  |
| Donega (2013) | X |  |  |  |  | X |  |  |  |  |  |
| Donega (2014) | X |  |  |  |  |  |  |  |  |  |  |
| Donega (2015) | X |  |  |  |  | X |  |  |  |  |  |
| Gu (2015) |  |  |  |  | X |  |  |  |  |  |  |
| Gu (2016) |  |  |  |  | X | X |  |  |  |  | X |
| Jellema (2013) |  |  |  |  |  |  |  |  |  | X |  |
| Kim (2012) | X | X |  |  |  |  |  |  |  |  |  |
| Lee (2010) | X | X |  |  |  |  |  |  |  |  |  |
| van Velthoven (2010)A | X |  |  |  |  |  |  |  |  |  |  |
| van Velthoven (2010)B | X | X |  |  |  |  |  |  |  |  |  |
| van Velthoven (2010)C | X |  |  |  |  |  |  |  |  |  |  |
| van Velthoven (2012) | X |  |  |  |  |  |  |  |  |  |  |
| van Velthoven (2013) | X |  |  | X |  |  |  |  |  |  |  |
| Xia (2010) |  |  |  |  |  |  |  | X |  |  |  |
| Zhang (2014) |  | X |  |  | X |  |  |  | X |  |  |
| Zhou (2015) |  |  |  |  | X | X |  |  |  |  | X |
| Zhu (2014) | X |  |  |  |  |  | X |  |  |  |  |
