# Supplementary material for: Therapeutic potential of mesenchymal stromal cells for hypoxic ischemic encephalopathy: A systematic review and meta-analysis of preclinical studies
Source: PLoS One. 2017 Dec 19;12(12):e0189895. doi: 10.1371/journal.pone.0189895 (PMC5736208; doi:10.1371/journal.pone.0189895)
Supplement: S5 Table — (DOCX) [file pone.0189895.s006.docx]

**Supplementary Table 5.** MSC criteria reported by included studies

| **Author (Year)** | **Does the study report MSC criteria were met?** | **Were MSCs purchased or supplied?** | **Plastic adherence?** | **Positive markers** | **Negative markers** | **Differentiation capability** | **Cell expansion media** | **Passage number** |
| --- | --- | --- | --- | --- | --- | --- | --- | --- |
| Cameron (2015) | Yes | No | Yes | CD 29, CD 90 | CD 34, CD 45 | Not reported | DMEM,  10% FBS, Penicillin, Streptomycin | 3 to 5 |
| Ding (2014) | Yes | No | Yes | CD 29, CD 44, CD 90, CD 105 | CD 45 | Adipocytes, Osteocytes | Alpha-MEM, 10% FBS, Penicillin, Streptomycin | 4 to 5 |
| Donega (2013) | No | Invitrogen | Not reported | Not reported | Not reported | Not reported | Not reported | Not reported |
| Donega (2014) | Yes | No | Yes | CD 73, CD 90, CD 105 | CD 45 | Not reported | Alpha-MEM, Human platelet lysate | 3 |
| Donega (2015) | No | Invitrogen | Not reported | CD 73, CD 90, CD 105 | CD 45 | Not reported | Not reported | Not reported |
| Gu (2015) | No | No | Not reported | Not reported | Not reported | Not reported | DMEM/F12, 10% FBS | Not reported |
| Gu (2016) | No | No | Not reported | Not reported | Not reported | Not reported | Not reported | Not reported |
| Jellema (2013) | Yes | Merck Millipore | Not reported | HLA-I, CD 13, CD 44, CD 49c, CD 54, CD 58, CD 73, CD 90, CD 105, CD 140b | HLA-II, CD 3, CD 19, CD 34, CD 40, CD 45, CD 80, CD 86, CD 146 | Adipocytes, Osteocytes | DMEM/F12, 10% FBS | 4 |
| Kim (2012) | Yes | No | Yes | Oct-4, SSEA-4, HLA-AB, CD 73, CD 105 | HLA-DR, CD 14, CD 34, CD 45 | Adipocytes, Osteocytes, Chondrocytes Respiratory epithelium | Not reported | 5 |
| Lee (2010) | Yes | No | Yes | CD 73, CD 105 | CD 14, CD 34, CD 45 | Not reported | 10% DMEM-low glucose, 10% FBS, 1% antibiotic-antimycotic solution | <5 |
| van Velthoven (2010)A | Yes | Jackson Laboratories | Yes | Sca-1, MHC-I, CD 29, CD 44, CD 90 | Myeloid and hematopoietic cell lineage specific antigens | Adipocytes, Osteocytes, Chondrocytes | DMEM, 15% FBS | Not reported |
| van Velthoven (2010)B | Yes | Jackson Laboratories | Yes | Sca-1, MHC-I, CD 29, CD 44, CD 90 | Myeloid and hematopoietic cell lineage specific antigens | Not reported | DMEM, 15% FBS | Not reported |
| van Velthoven (2010)C | Yes | Jackson Laboratories | Yes | Sca-1, MHC-I, CD 29, CD 44, CD 90 | Myeloid and hematopoietic cell lineage specific antigens | Not reported | DMEM, 15% FBS | Not reported |
| van Velthoven (2012) | Yes | Jackson Laboratories | Yes | Sca-1, MHC-I, CD 29, CD 44, CD 90 | Myeloid and hematopoietic cell lineage specific antigens | Not reported | DMEM, 15% FBS | Not reported |
| van Velthoven (2013) | Yes | GIBCO | Yes | CD 29, CD 44, CD 90, CD 106 | Myeloid and hematopoietic cell lineage specific antigens | Not reported | DMEM | Not reported |
| Xia (2010) | Yes | No | Yes | CD 29, CD 44, CD 105 | CD 34, CD 45 | Adipocytes, Osteocytes, Chondrocytes | DMEM, 20% FBS, Penicillin Streptomycin,  L-glutamine | 10 |
| Zhang (2014) | Yes | No | Yes | CD 73, CD 105, CD 90 | CD 14, CD 34, CD 45, CD 79a | Fibroblasts | StemPro MSC-free medium, 10% FBS, Penicillin, Streptomycin | 3 |
| Zhou (2015) | Yes | Chongqing Stem Cell Bank | Yes | HLA-ABC,  CD 29, CD 44, CD 90, CD 105 | HLA-DR, CD 34, CD 45 | Not reported | DMEM/F12, 10% FBS, Penicillin, Streptomycin | 5 to 10 |
| Zhu (2014) | No | Stem Cell Engineering Research Center | Not reported | Not reported | Not reported | Multipotent phenotypes | DMEM, 15% FBS | Not reported |
| **Abbreviations:** Dulbecco Modified Eagle Medium (DMEM); fetal bovine serum (FBS); Modified Eagle Medium (MEM) | | | | | | | | |
