# Supplementary material for: Therapeutic potential of mesenchymal stromal cells for hypoxic ischemic encephalopathy: A systematic review and meta-analysis of preclinical studies
Source: PLoS One. 2017 Dec 19;12(12):e0189895. doi: 10.1371/journal.pone.0189895 (PMC5736208; doi:10.1371/journal.pone.0189895)
Supplement: S6 Table — (DOCX) [file pone.0189895.s007.docx]

**Supplementary Table 6.** SYRCLE Risk of Bias Assessment for included studies

| **Author (Year)** | **Random sequence generation?** | **Groups similar at baseline?** | **Allocation concealed?** | **Animals randomly housed?** | **Blinding of caregivers and/or examiners?** | **Random selection for outcome assessment?** | **Blinding of outcome assessor?** | **Incomplete outcome data addressed?** | **Free from selective outcome reporting?** | **Free from other bias?** |
| --- | --- | --- | --- | --- | --- | --- | --- | --- | --- | --- |
| Cameron (2015) | Unclear | Yes | Unclear | Unclear | Unclear | Unclear | Yes | Yes | Yes | Yes |
| Ding (2014) | Unclear | Yes | Unclear | Unclear | Unclear | Unclear | Unclear | Yes | Yes | Yes |
| Donega (2013) | Unclear | Yes | Unclear | Unclear | Unclear | Unclear | Unclear | Yes | Yes | Yes |
| Donega (2014) | Unclear | Yes | Unclear | Unclear | Unclear | Unclear | Unclear | Yes | Yes | Yes |
| Donega (2015) | Unclear | Yes | Unclear | Unclear | Unclear | Unclear | Unclear | Yes | Yes | Yes |
| Gu (2015) | Unclear | Yes | Unclear | Unclear | Unclear | Unclear | Unclear | Yes | Yes | Yes |
| Gu (2016) | Unclear | Yes | Unclear | Unclear | Unclear | Unclear | Unclear | Yes | Yes | Yes |
| Jellema (2013) | Unclear | Yes | Yes | Unclear | Yes | Yes | Yes | Yes | Yes | Yes |
| Kim (2012) | Unclear | Yes | Unclear | Unclear | Yes | Unclear | Yes | Yes | Yes | Yes |
| Lee (2010) | Unclear | Yes | Unclear | Unclear | Unclear | Unclear | Yes | Yes | Yes | Yes |
| van Velthoven (2010)A | Unclear | Yes | Unclear | Unclear | Unclear | Unclear | Unclear | Yes | Yes | Yes |
| van Velthoven (2010)B | Unclear | Yes | Unclear | Unclear | Unclear | Unclear | Unclear | Yes | Yes | Yes |
| van Velthoven (2010)C | Unclear | Yes | Unclear | Unclear | Unclear | Unclear | Unclear | Yes | Yes | Yes |
| van Velthoven (2012) | Unclear | Yes | Unclear | Unclear | Unclear | Unclear | Unclear | Yes | Yes | Yes |
| van Velthoven (2013) | Unclear | Yes | Unclear | Unclear | Unclear | Unclear | Unclear | Yes | Yes | Yes |
| Xia (2010) | Unclear | Yes | Unclear | Unclear | Unclear | Unclear | Yes | Yes | Yes | Yes |
| Zhang (2014) | Unclear | Yes | Unclear | Unclear | Unclear | Unclear | Unclear | Yes | Yes | Yes |
| Zhou (2015) | Unclear | Yes | Unclear | Unclear | Unclear | Unclear | Unclear | Yes | Yes | Yes |
| Zhu (2014) | Unclear | Yes | Unclear | Unclear | Unclear | Unclear | Unclear | Yes | Yes | Yes |
