# Supplementary figures and images for: Therapeutic potential of mesenchymal stromal cells for hypoxic ischemic encephalopathy: A systematic review and meta-analysis of preclinical studies
Source: PLoS One. 2017 Dec 19;12(12):e0189895. doi: 10.1371/journal.pone.0189895 (PMC5736208; doi:10.1371/journal.pone.0189895)

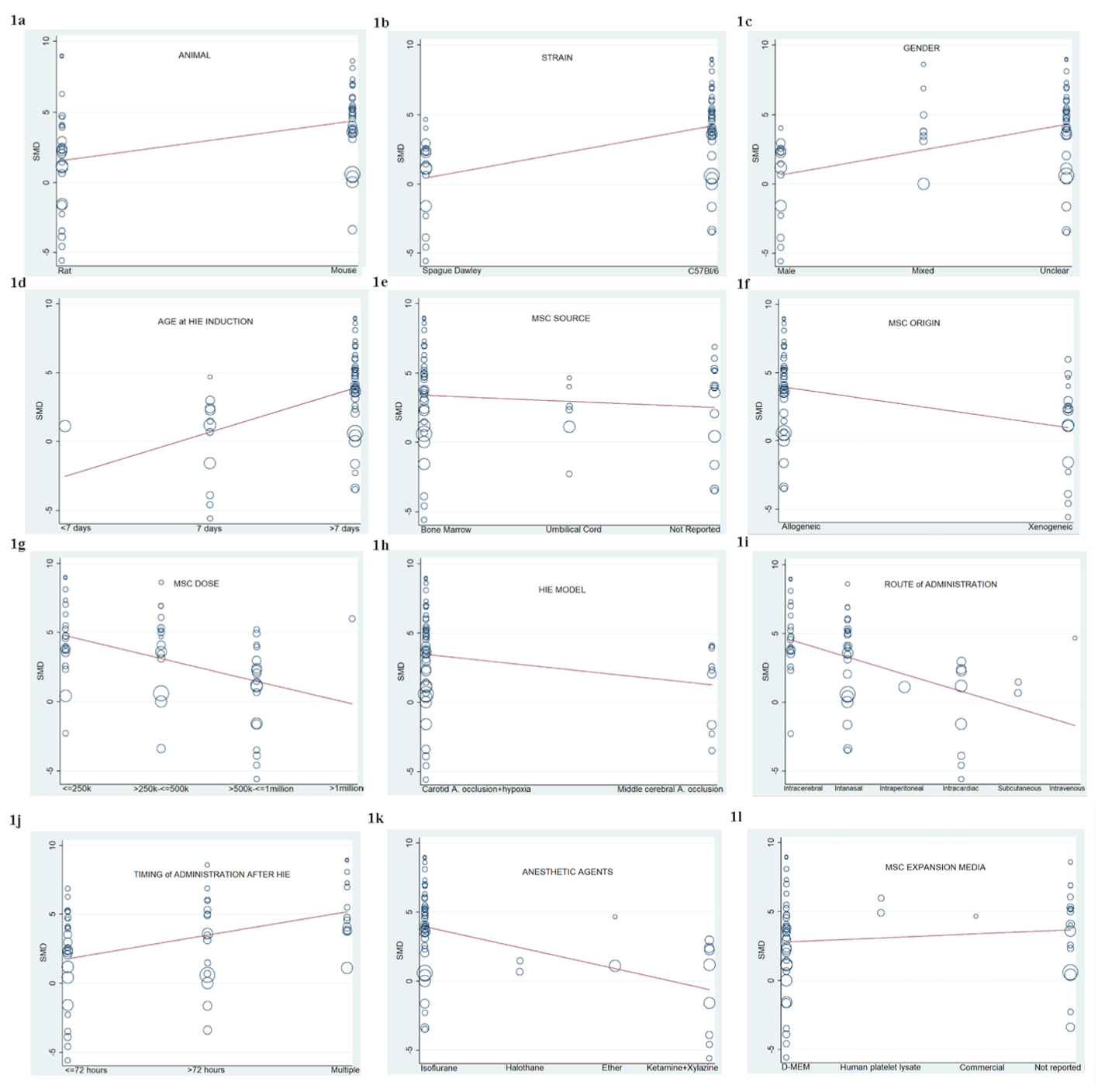

Supplement: S1 Fig — (a) animal, (b) strain, (c) gender, (d) age at HIE induction, (e) MSC source, (f) MSC origin, (g) MSC dose, (h) HIE model, (i) route of administration, (j) timing of administration, (k) anesthetic agents, (l) MSC expansion media. (TIFF) [file pone.0189895.s008.tiff]

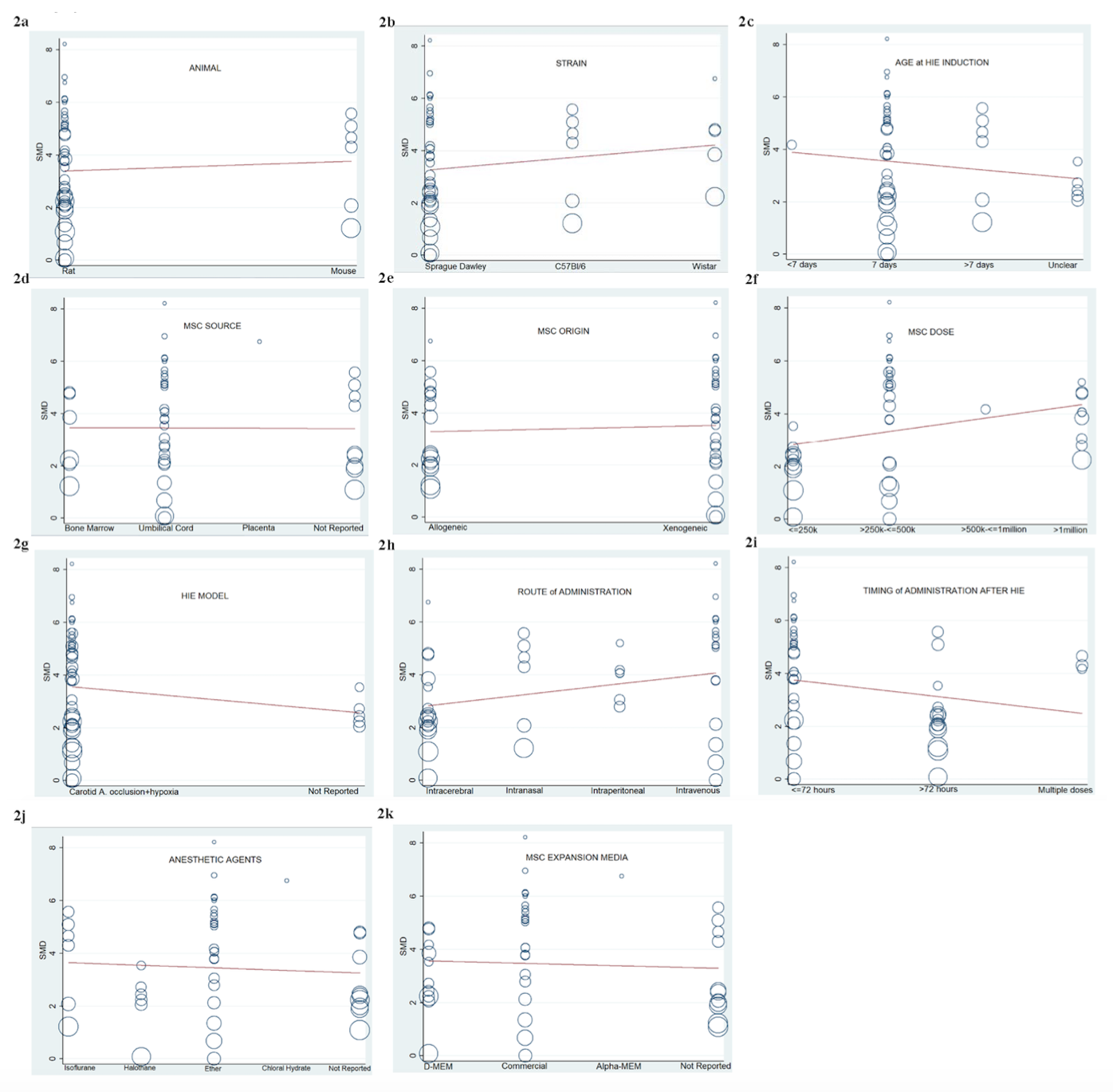

Supplement: S2 Fig — (a) animal, (b) strain, (c) age at HIE induction, (d) MSC source, (e) MSC origin, (f) MSC dose, (g) HIE model, (h) route of administration, (i) timing of administration, (j) anesthetic agents, (k) MSC expansion media. (PNG) [file pone.0189895.s009.png]
